# The effect of on-demand vs deep neuromuscular relaxation on rating of surgical and anaesthesiologic conditions in patients undergoing thoracolaparoscopic esophagectomy (DEPTH trial): study protocol for a randomized controlled trial

**DOI:** 10.1186/s13063-015-0849-0

**Published:** 2015-08-05

**Authors:** Denise P. Veelo, Suzanne S. Gisbertz, Rebekka A. Hannivoort, Susan van Dieren, Bart F. Geerts, Mark I. van Berge Henegouwen, Markus W. Hollmann

**Affiliations:** Department of Anaesthesiology, Academic Medical Center, Meibergdreef 9, Amsterdam, 1105 AZ The Netherlands; Department of Surgery, Academic Medical Center, Meibergdreef 9, Amsterdam, 1105 AZ The Netherlands

**Keywords:** Surgical rating scale, Neuromuscular relaxation, Anaesthesia, Minimally invasive esophagectomy

## Abstract

**Background:**

Deep muscle relaxation has been shown to facilitate operating conditions during laparoscopic surgery. Minimally invasive esophageal surgery is a high-risk procedure in which the use of deep neuromuscular block (NMB) may improve conditions in the thoracic phase as well. Neuromuscular antagonists can be given on demand or by continuous infusion (deep NMB). However, the positioning of the patient often hampers train-of-four (TOF) monitoring. A continuous infusion thus may result in a deep NMB at the end of surgery. The use of neostigmine not only is insufficient for reversing deep NMB but also may be contraindicated for this procedure because of its cholinergic effects. Sugammadex is an effective alternative but is rather expensive. This study aims to evaluate the use of deep versus on-demand NMB on operating, anaesthesiologic conditions, and costs in patients undergoing a two- or three-phase thoracolaparoscopic esophageal resection.

**Methods/Design:**

We will conduct a single-center randomized controlled double-blinded intervention study. Sixty-six patients undergoing a thoracolaparoscopic esophageal resection will be included. Patients will receive either continuous infusion of rocuronium 0.6 mg/kg per hour (group 1) or continuous infusion of NaCl 0.9 % 0.06 ml/kg per hour (group 2). In both groups, on-demand boluses of rocuronium can be given (open-label design).

The primary aim of this study is to compare the surgical rating scale (SRS) during the abdominal phase. Main secondary aims are to evaluate SRS during the thoracic phase, to evaluate anesthesiologic conditions, and to compare costs (in euros) associated with use of rocuronium, sugammadex, and duration of surgery.

**Discussion:**

This study is the first to evaluate the benefits of deep neuromuscular relaxation on surgical and anaesthesiologic conditions during thoracolaparoscopic esophageal surgery. This surgical procedure is unique because it consists of both an abdominal phase and a thoracic phase taking place in different order depending on the subtype of surgery (a two- or three-stage transthoracic esophagectomy). In addition, possible benefits associated with deep NMB, such as decrease in operating time, will be weighed against costs.

**Trial registration:**

European Clinical Trials Database (EudraCT) number: 2014-002147-18 (obtained 19 May 2014)

ClinicalTrials.gov: NCT02320734 (obtained 18 Dec. 2014)

## Background

Deep muscle relaxation has been shown to facilitate surgical field visibility and operating conditions as measured by the surgical rating scale (SRS) during laparoscopic surgery [1–5]. However, most of these studies were short-lasting low-risk procedures such as laparoscopic cholecystectomy. Minimally invasive thoracolaparoscopic esophageal surgery is a high-risk procedure in which the use of deep neuromuscular block (NMB) may also increase field visibility and improve anaesthesia conditions such as straining, high peak insufflation pressures, and respiratory and cardiac incidents [1–5]. However, the positioning of the patient (prone and supine with both arms alongside the body) during this procedure often hampers accurate train-of-four (TOF) measurement to evaluate the depth of the NMB. During these surgical procedures, boluses of muscle relaxants are often given randomly or on indication only, at the discretion of the surgeon or anaesthesiologist. Alternatively, continuous infusion of rocuronium can be used but this might result in a deep NMB at the end of surgery, leading to a prolonged emergence from anaesthesia. To reduce post-operative respiratory complications, but also to facilitate rapid extubation of the trachea rather than post-operative mechanical ventilation in the intensive care unit (ICU), neuromuscular antagonists are often needed.

The use of neostigmine not only is insufficient for reversing deep NMB but also is contraindicated for moderate NMB because of its cholinergic effects on smooth muscle that may increase the risk of anastomotic dehiscence, although the evidence is conflicting [6–8]. In addition, neostigmine may be associated with increased risk of respiratory complications post-operatively because of high incidence of residual neuromuscular relaxation [9]. Sugammadex is a safe and effective alternative to neostigmine but is rather expensive. Based on the high costs of sugammadex, the administration of rocuronium on indication only may be preferred over a continuous deep muscle relaxation, despite the lack of reliable TOF monitoring to ensure adequate depth of relaxation at all times during the procedure. The thoracolaparoscopic esophageal resection consists of two or three phases: an abdominal phase and a thoracic phase and a cervical phase if indicated. Accordingly, depending on the location of the anastomosis, the procedure will start in a prone (thoracic phase) or a supine (abdominal phase) position. Whether the abdominal phase takes places before or after the thoracic phase may influence SRS scores and has never been evaluated. It is uncertain whether a deep NMB has the same advantages during thoracic surgery as during abdominal surgery, because of the rigidity of the ribcage, which keeps the surgical field open. This double-blind randomized controlled study aims primarily to evaluate the use of two muscle relaxant regimes on operating (SRS) and anaesthesiologic conditions in patients undergoing thoracolaparoscopic esophageal resection.

## Methods

The protocol of this study was approved by the local ethics committee (Academic Medical Center, registration number 2014_211#B20141036) and registered at ClinicalTrials.gov under number NCT02320734. The study will be conducted in accordance with the principles of the Declaration of Helsinki in the current version of Fortaleza, Brazil 2013 and in accordance with the Medical Research Involving Human Subjects Act (WMO).

### Design

This study is designed as a single-center randomized controlled double-blinded trial. Patients are randomly assigned to receive either continuous infusion of rocuronium 0.6 mg/kg per hour (group 1) or continuous infusion of NaCl 0.9 % 0.06 ml/kg per hour (group 2). This is started immediately after induction. Treatment allocation will be done in a double-blinded fashion (including both the surgeon and the anaesthesiologist), to standard (on indication) versus deep NMB. Blinding will be done by a computerized randomization code. We will randomly assign according to the principle of balanced assignment: the order in which patients are classified to the different groups will be determined in a computer-randomized order. Block randomization will be done and stratified by type of surgery (start in supine or prone position) to correct for any influence of this parameter on the study endpoints. The reasons for withdrawal will be accurately documented in the case report forms. The medication is prepared and labeled by the pharmacy and supplied by the (blinded) researcher to the blinded anaesthesiologist in charge of the patient. Double blinding will be maintained during the study unless the anaesthesiologist in charge needs to unblind the data in case of a medical emergency.

### Patients

Sixty-six patients will be included. Inclusion and exclusion criteria are presented in Table 1. Patients are included only after informed consent is obtained.

**Table 1:** Study in- and exclusion criteria

Inclusion criteria	Exclusion criteria
Age of at least 18 years	Age less than 18 or pregnancy
Elective thoracolaparoscopic esophageal resection (either two- or three-stage approach)	Known allergies for aminosteroid-type muscle relaxants or sugammadex
Written informed consent	Severe kidney dysfunction (glomerular filtration rate of less than 30), patients on dialysis
	Liver function disorders
	Myasthenia gravis or other (neuro)muscular diseases
	Carcinomatosis
	Use of anti-epileptica and lithium or drugs containing Kinine

### Study procedures and measurements

Prior to the start of the study, both surgeons will be trained in rating, by practicing the SRS during two procedures, containing about 32 SRS scores. During surgery, operating conditions will be evaluated every 30 min for both the abdominal and thoracic phases by using a 5-point SRS ranging from 1 (poor surgical conditions, intervention necessary) to 5 (optimal surgical conditions) as well as a continuous numeric rating scale ranging from 1 to 100 (Table 2) [2]. Scoring will be done by the two surgeons performing the procedure (SG and MB). One SRS and one numeric rating score are registered each 30 min (with a variation of 31–40 min if the surgeon’s attention is needed elsewhere). If both surgeons are operating on the same patient, agreement on the score must be reached.

**Table 2:** Surgical rating scale

1	*Extremely poor conditions*: the surgeon is unable to work because of coughing or because of the inability to obtain a visible laparoscopic field because of inadequate muscle relaxation. Additional neuromuscular blocking agents must be given
2	*Poor conditions*: there is a visible laparoscopic field, but the surgeon is severely hampered by inadequate muscle relaxation with continuous muscle contractions, movements, or both with the hazard of tissue damage. Additional neuromuscular blocking agents must be given
3	*Acceptable conditions*: there is a wide visible laparoscopic field, but muscle contractions, movements, or both occur regularly, causing some interference with the surgeon’s work. There is the need for additional neuromuscular blocking agents to prevent deterioration
4	*Good conditions*: there is a wide laparoscopic working field with sporadic muscle contractions, movements, or both. There is no immediate need for additional neuromuscular blocking agents unless there is the fear of deterioration
5	*Optimal conditions*: there is a wide visible laparoscopic working field without any movement or contractions. There is no need for additional neuromuscular blocking agents

Surgical rating scale adapted from Martini et al. [2]

If indicated by either surgeon or anaesthesiologist, all patients, irrespective of their group assignment, can receive boluses of open-label rocuronium 0.30 mg/kg. The reason for the on-demand bolus is registered. Surgical conditions will be scored immediately before and 5 min after a bolus when requested by the surgeon only, so as not to affect blinding of the surgeons. Indications for bolus infusion are high peak inflation pressure (>35 mm Hg), high abdominal (>15 mm Hg) and thorax (>8 mm Hg) insufflation pressure, and movement, breathing, or straining of the patient. Intra-abdominal pressure will be registered every 30 min from the abdominal CO_2_ insufflation device.

For neuromuscular function monitoring, the TOF-watch SX acceleromyograph (MSD BV, Haarlem, The Netherlands) will be used. During the procedure, a TOF count (situated at the flexor hallucis brevis of the leg) will be done every 30 min at the same time as the evaluation of the surgical conditions and directly before and 5 min after an on-demand bolus [10]. Both the anaesthesiologist and the surgeon will be blinded for the results of this measurement.

When the operation is finished, a TOF count and ratio are done at the ulnar nerve (not blinded). Depth of NMB will be scored as follows: (1) very deep NMB (post-tetanic count, no twitches, (2) deep NMB (post-tetanic count, one or two twitches), (3) moderate NMB (TOF, one or two twitches), and (4) shallow or no NMB (TOF four twitches, ratio of more than 0 %). In patients with a very deep or deep NMB, a TOF is repeated 30 min after infusion of sugammadex to check for recurarization. Based on the TOF count at the end of operation, sugammadex is given at a dose of 16 mg/kg in very deep block, 4 mg/kg in deep NMB, 2 mg/kg in moderate NMB, and 2 mg/kg in shallow NMB. If recurarization is found, an additional dose of sugammadex is given on the basis of the TOF count found at that time. In case of a clinical reason to continue mechanical ventilation after surgery, administration of sugammadex is postponed until one post-tetanic twitch is found. The sugammadex dose needed in case of a very deep block (16 mg/kg) was added to the protocol after 10 patients.

### Drug accountability and safety evaluations

Preparation of study drugs will be performed on the basis of authorised drug preparation protocols by the hospital pharmacy. After preparation, the syringes will be labelled according to local guidelines and dispensed to research staff only; no additional labelling will be performed.

The investigator will be responsible for the destruction of the supplies at the study center pursuant to the ICH/GCP (International Conference on Harmonisation of Technical Requirements for Registration of Pharmaceuticals for Human Use/Good Clinical Practice) Guidelines, local regulations, and the investigator’s institutional policies. Clinical supplies will be received by the Academic Medical Center hospital pharmacy at the study site, handled and stored safely and properly, and kept in a secured location with limited access. Clinical supplies are dispensed in accordance with the protocol. The investigator is responsible for keeping accurate records of the clinical supplies, the amount dispensed to and returned by the patients, and the disposition at the end of the study. We will conform with the requirement for adverse experience reporting according to Good Clinical Practice guidelines and local Medical Ethics Committee practice.

### Outcome parameters

#### Primary parameter

The primary parameter is rating of surgical conditions (SRS), as described in Table 2, during the abdominal phase of the operation [2].

#### Secondary parameters

- Rating of SRS during thoracic phase of the operation, number of on-demand boluses infused, and indication of on-demand bolus administration.
- Rating of anaesthesia conditions, including peak and mean respiratory pressures and incidence peak insufflation pressure of more than 35 mm Hg; depth of NBM at the end of surgery, intraoperative cardiac and respiratory incidents; and time until spontaneous breathing and time until extubation after interruption of sedation.
- Costs: amount of rocuronium used (milligrams), dose of sugammadex needed (milligrams) to reach a TOF of more than 90 %, time to reversal, number of patients (percentage) with recurarization measured 30 min after reversal and consequently the need for additional sugammadex, and duration of surgery and operating room occupancy (expressed in hours and personnel cost per hour) and ICU stay.
- Perioperative surgical complications.
- The ability of surgeons and anaesthesiologists to estimate post-operatively to which group the patient was randomly assigned (at the end of each of the two phases).

#### Exploratory parameters

Outcome parameters are ICU/post-anaesthesia care unit (ICU/PACU) and hospital length of stay, need for re-intubation, post-operative surgical complications (bleeding, anastomotic leakage or bowel perforation, wound infections or sepsis, or re-operations), and pulmonary complications (pneumonia, pneumothorax, pleural effusion, or atelectasis). Pain numerical rating scores will be collected until dismissal ICU/PACU with a maximum of 24 h post-operatively.

These parameters are collected by means of registration forms during the procedure and on the following day. All other data will automatically be recorded by our Patient Database Management System. Hemodynamic monitoring will consist of stroke volume; stroke volume variation; heart rate; diastolic, systolic, and mean arterial pressure; systemic vascular resistance; central venous pressure; cardiac output; and cardiac index (EV1000; Edwards, Irvine, CA, USA). In addition, peripheral capillary oxygen saturation (SpO_2_), end-tidal carbon dioxide (etCO_2_), insufflation pressure, and mean airway pressure will automatically be recorded. Post-operative surgical complications will be prospectively collected.

### Patient flow chart

**Fig. 1:**
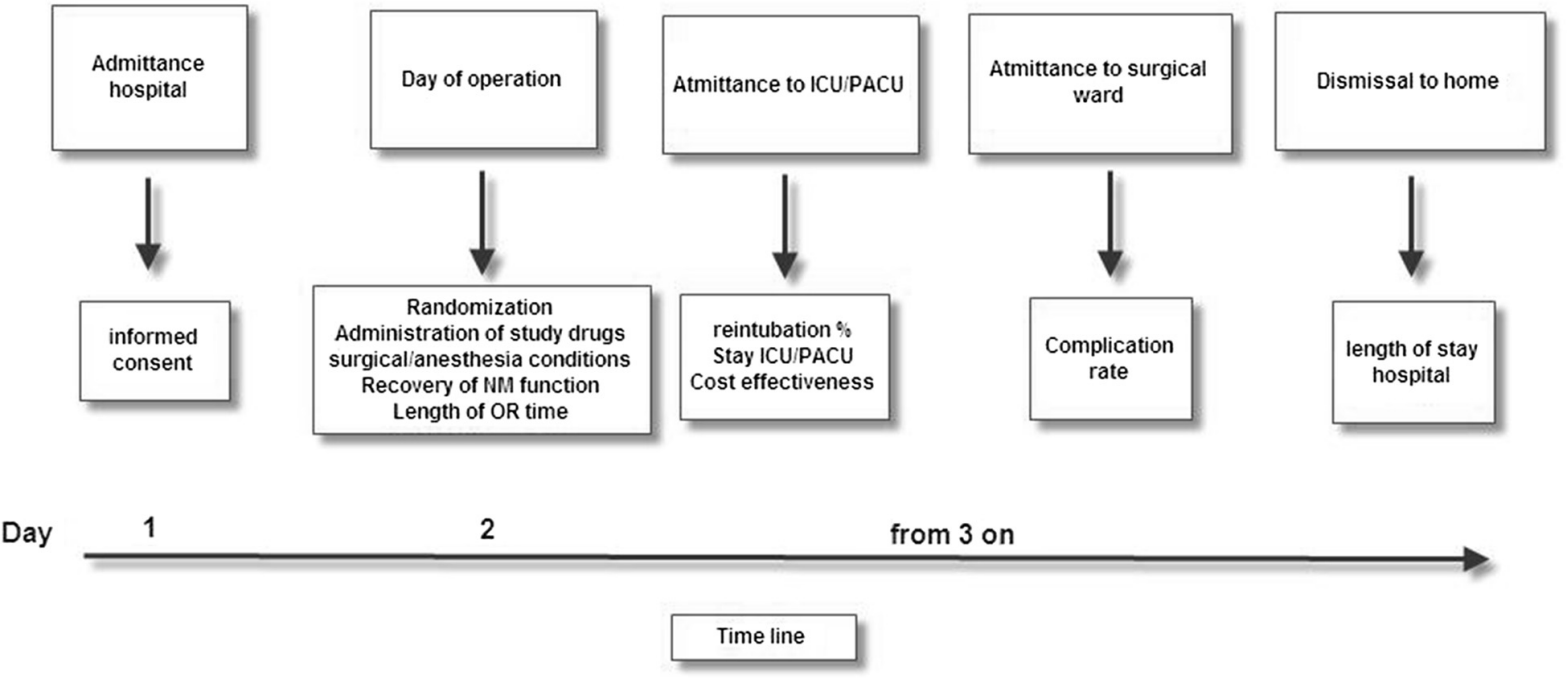
Study timeline. The different steps of the study and registrations over time, starting with admittance of the patient to the hospital until dismissal to home, are shown. Informed consent forms and study information are supplied to the patient before admittance. The signed forms are retrieved at the day of admittance. *ICU/PACU* intensive care unit/post-anaesthesia care unit, *NM* neuromuscular, *OR* operating room

Figure 1 shows the different phases in data collection during this study in a timeline.

### Other anaesthesia procedures

During the study, patients receive standard of care pre-operatively and post-operatively. The surgical procedure will not be changed. Also, induction of anaesthesia, the use of opioids, blood pressure treatment, fluid infusion, and mechanical ventilation will be standard of care.

#### Preparations

Patients will remain fasted for solid foods for 6 h and clear fluids for 2 h. Two hours prior to surgery, two sachets of preOp drink (Nutricia, Dublin, Ireland) or lemonade are given. Blood is drawn for blood type and cross-match analysis. A cannula is inserted into a vein on the right or preferably left hand or lower arm.

Prior to the operation, the patient is transported to the operating room, where a thoracic epidural is inserted with an antiseptic technique in a sitting position. The skin is anaesthetized with lidocaine 2 %, and the Tuohy needle is positioned with loss of resistance or hanging drop technique at a level of Th 6–7 or both.

#### Induction of anaesthesia

In the operating room, general anaesthesia is induced with propofol (approximately) 2–3 mg/kg, sufentanil for analgesia and 1.0 mg/kg rocuronium for paralysis. The trachea is intubated and the lungs are mechanically ventilated with pressure-regulated volume control.

After induction, general anaesthesia is maintained with sevoflurane at a minimal alveolar concentration of 1 and, when needed (on the basis of signs of pain), additional boluses of sufentanil. An arterial line (into the right or left brachial artery), a right subclavian tri-lumen central line, and a double-lumen gastric tube are placed. A urinary catheter is inserted (suprapubic in men). The epidural catheter is loaded with 10 ml bupivacaine 0.25 % in two tempi. One hour later, a continuous infusion of bupivacaine 0.25 % is started (0.08–0.1 ml/kg per hour). In case of a failed epidural, intravenous (i.v.) ketamine (bolus 0.25 mg/kg, maintenance 0.1 mg/kg) continuously is started in combination with i.v. sufentanil continuously 0.5–1 mcg/kg per hour. Cefazoline (Kefzol®) 2 g and metronidazol (Flagyl®) 500 mg are given prior to incision. Cefazoline is repeated according to the national antibiotic guidelines.

#### Surgical procedure

##### Two-stage thoracolaparoscopic esophagectomy (Ivor Lewis procedure)

The patient is placed in a supine position. A laparoscopy is performed by using 5 trocars and a maximum pressure of 15 mm Hg. During laparoscopy, a lymphadenectomy is performed (hepatoduodenal ligament, common hepatic artery, celiac trunk, splenic artery, splenic hilum, paracardial left and right), the greater curvature is mobilized, identifying and sparing the right gastro-epiploic vessels, and a gastric tube (3 cm wide) is created by using a linear stapling device. A jejunostomy catheter is placed approximately 20 cm distal of Treitz ligament, the abdominal phase is terminated, and the incisions are closed (fascia and skin).

Next, the patient is repositioned to a prone position. A thoracoscopy is performed by using 4 trocars and a maximum insufflation pressure of 6–8 mm Hg. The thoracic esophagus is mobilized, and a lymphadenectomy is performed (stations 4, 5, 7, 8, 9, and 10 according to the American Joint Committee on Cancer classification for esophageal cancer). The arch of the azygos vein is divided by using a vascular stapling device. The thoracic duct is transected at the level of the diaphragm and arch of the azygos vein by using 10-mm endoclips and excised with the specimen. The esophagus is divided just cranial to the level of the arch of the azygos vein. The specimen and gastric tube are retrieved in the thorax. A minithoracotomy (5 cm) is performed through which the specimen is resected. An anastomosis is created by using a circular stapling device. The anastomosis may be subsequently sutured with interrupted Vicryl 3.0 sutures. The anastomosis is concealed under the pleura, and an omental wrap is placed around the anastomosis. A nasogastric tube is placed in the gastric tube. After placement of a thoracic drain, the thoracoscopy wounds are closed (muscles and skin).

##### Three-stage thoracolaparoscopic esophagectomy (McKeown procedure)

If a subtotal esophagectomy with high paratracheal lymph node dissection (including station 2) is indicated, a three-stage procedure will be performed. A thoracoscopy is performed first, followed by a laparoscopy. Eventually, a left cervical incision is performed on the ventral border of the sternocleidomastoid muscle, and the cervical esophagus is mobilized and transected with attention for the recurrent laryngeal nerve on both sides. The specimen is retrieved intra-abdominally and removed. The gastric tube is created and pulled up through the mediastinum, and a cervical end-to-end anastomosis is created by using PDS 3.0 intermittent sutures. A nasogastric tube is positioned in the gastric tube. The cervical incision is closed with an intracutaneous suture.

#### Post-operative care

Depending on American Society of Anesthesiologists (ASA) classification, patients will go either to the PACU (ASA 1 or 2) or to the ICU (ASA 3 or higher). The endotracheal tube will be removed as soon as possible after stopping sedation, either in the operating theatre or after arrival in PACU/ICU. Numerical pain rating—on a scale from 0 (no pain) to 10 (most severe pain imaginable)—will be collected each hour.

### Sample size calculation

The sample size is based on an article by Martini et al. [2], who showed a difference of 0.7 points on the SRS and a standard deviation (SD) of 0.4. However, compared with the retroperitoneal surgery studied in that article, the current procedure of esophagectomy is anticipated to have more sources of variability for the surgical conditions. This may reduce the benefit or effect size of treatment to some degree but in particular will increase the variability, even when adjusting for body mass index and the presence of previous procedures, as other factors cannot be effectively controlled. Table 3 shows a range of plausible effect sizes and (adjusted) SDs of the SRS. A sample size of 60 in total would provide a power of 90 % to show a mean difference of 0.6 with a standard deviation of 0.7 and a two sided alpha level of 0.05. This mean difference is slightly lower than observed in the study by Martini et al of 0.6 points on the SRS and a somewhat increased SD of 0.7 points on the SRS. A drop-out of 10 % is expected and therefore we will include 66 patients. After withdrawal of informed consent before the operation but after randomization, the subject will be replaced by a new patient.

**Table 3:** Range of plausible effect sizes and (adjusted) standard deviations of the SRS

Mean group difference	Standard deviation			
	0.5	0.6	0.7	0.8
0.5	23	32	43	55
0.6	16	23	30	39
0.7	12	17	23	29

Sample size per group by a range of mean group differences and standard deviations (SDs) on the surgical rating scale (SRS) at 90 % power. A sample size of 60 in total would provide a power of 90 % in case the effect size is slightly lower as observed in Martini et al. [2] of 0.6 points on the SRS and a somewhat increased SD of 0.7 points on the SRS. A drop-out of 10 % is expected and therefore we will include 66 patients

### Statistical analysis

The data analysis will be based on an intention-to-treat approach. Baseline continuous variables will be summarized by using mean with SD or median with interquartile range as appropriate. Categorical variables will be summarized by using percentage and number of patients. The SRS will be measured repeatedly. These repeated measurements will be summarized to a mean for every patient. These values will follow a normal distribution and therefore be tested by using a Student’s *t* test. A two-sided *P* value of less than 0.05 will be considered statistically significant. The difference in SRS between the two groups will further be examined by using a general linear model. Covariates will be analyzed univariately. Covariates will be included in a multivariable analysis if the univariate *P* value is less than 0.20. A backward selection process will be used to achieve the most optimal model.

Covariates to be considered are anaesthesia- or surgery-related (for example, timing of abdominal and thoracic phase, drug dosages, use of epidural anaesthesia, and duration of surgery) and patient-related (for example, surgical history, age, weight, and body mass index). Patients with less than three surgical rating scores because of early termination of the surgical procedure will be excluded in a secondary analysis. Additional analyses will be performed on the secondary outcome parameters. No interim analysis will be done; the study will be terminated after enclosure of the last patient. Because of the amendment in sugammadex dose, the first 10 patients will be excluded for the cost analysis only. A sensitivity analysis, including the first 10 patients, will be performed.

## Discussion

This study is the first to evaluate the benefits of deep neuromuscular relaxation during thoracolapascopic esophageal surgery. In addition, this type of surgery is unique in that it consists of both an abdominal and a thoracic phase and because of its long duration, enabling acquisition of an adequate number of SRS scores. Considering the results of earlier studies, we can expect a benefit in operating conditions of deep neuromuscular relaxation during the abdominal phase. However, whether the operation is started with the abdominal or thoracic phase may influence SRS scores because of cumulative doses of anaesthetics in a later phase. Therefore, the benefit may be smaller when the abdominal phase comes last. To better determine this influence, patients are randomly assigned in blocks. Whether a benefit can be found during the thoracoscopic phase has yet to be determined. The ribcage will keep the surgical field open, and the diaphragm may be the only influence on surgical conditions. In addition, as this operation normally takes 8 h, deep neuromuscular relaxation may shorten duration of operation. However, this benefit may be counteracted by the costs of rocuronium and sugammadex and time until extubation. A cost analysis will be done to answer this question.

We will use the same scoring system as was developed by Martini et al. [2]. It showed adequate reliability in discrimination between surgical conditions and a low variability between surgeons. The scoring will be performed by two surgeons with ample experience with the procedure, and both are trained in SRS scoring before the start of the study. In contrast to Martini et al., we chose not to include the anaesthesiologist in the SRS scoring, because of lack of expertise in assessing the quality of the (laparoscopic/thoracoscopic) surgical field. Anaesthesiologists will assess the anaesthesia conditions (peak and mean respiratory pressures, incidence peak insufflation pressure of more than 35 mm Hg, depth of NBM at the end of surgery, intraoperative cardiac and respiratory incidents, and the indication for on-demand neuromuscular blocking agent).

The reasons for anaesthesiologists not to standardize a deep NMB regime may be the expected need for reversal at the end of the procedure. When employing deep NMB, one has the option to use sugammadex, which is costly, or have the patient on mechanical ventilation post-operatively, which may increase the incidence of pneumonia following surgery [1]. Reversal with (high-dose) neostigmine is usually not favored following these procedures in fear of the cholinergic effects on the newly formed anastomosis, although recent evidence for the adverse effects on anastomotic patency is scarce and the existing data are conflicting [6–8].

Sugammadex has been shown to be a safe and fast alternative to reverse the neuromuscular blocking induced by rocuronium, even when it was administered continuously for up to 5 h [11]. However, as yet, no data on the use of sugammadex after 8–9 h of deep (continuous) neuromuscular relaxation and the incidence of recurarization exist. Therefore, we will register time until TOF of more than 90 % and the incidence of recurarization.

Furthermore, lack of adequate neuromuscular monitoring (e.g., TOF)—in the prone position but especially in the supine position, where both hands are tucked in alongside the body—is challenging to the anaesthesiologist. Hand movements are often inhibited, and TOF counts are unreliable. In view of that, anaesthesiologists and surgeons often disagree about the operating conditions and the ability of the surgeons to judge whether the patient is adequately relaxed or not. We therefore let the surgeons (and anaesthesiologist) “estimate” at the end of the operation to which group the patient was randomly assigned.

### Limitations

This study has some unavoidable limitations instigated by the included surgical population and chosen design. During these procedures, TOF count is not reliable in prone positioning and supine positioning, because of the movement confinement of the body. Although this is part of the normal situation anaesthesiologists face during these procedures, we cannot be absolutely sure whether the patients randomly assigned to receive deep neuromuscular relaxation are insufficiently relaxed or too deeply relaxed. Indeed, for adequate neuromuscular relaxation of the diaphragm, a post-tetanic count of between 0 and 1 is probably necessary and this is very hard to accomplish in normal practice. We will use the standard dosing for continuous use that is advised in the product specifics (Summary of Product Characteristics text) of rocuronium and sugammadex. However, because of the lack of data on continuous administration for more than 5 h, there may be variability in NMB seen, and some patients may have a very deep block at the end of the procedure. We therefore amended the protocol after 10 patients, more clearly describing the management of patients with a very deep block at the end of the operation. In addition, we use the peroneal nerve to measure TOF because we expect less movement inhibition. However, it is possible that, owing to positional problems, we cannot get a reliable TOF for every patient. Consequently, we do not adjust the infused rocuronium dose on the basis of these measurements.

Another limitation is that the surgeons might guess in which group the patient is randomly assigned because of high abdominal pressures, respiratory events, or diaphragm movements. This might influence scoring of SRS. A recent study, however, showed that surgeons were not able to estimate the group assignment to deep versus moderate neuromuscular relaxation. They estimated correctly in a little more than 50 % and this can also be explained by chance. We also let the surgeons estimate group assignment to be able to estimate bias in SRS. In addition, we chose for a neuromuscular regime that is common practice (on demand) and compared that with a regime that is not (deep neuromuscular relaxation). Based on the results of the present study we will weigh benefits against costs.

## Trial status

Approval of the protocol has been obtained, and recruitment of the patients has started. The study end is planned in June 2016.

## Abbreviations

ASA: American Society of Anesthesiologists
ICU: Intensive care unit
i.v.: Intravenous
NMB: Neuromuscular block
PACU: Post-anaesthesia care unit
SD: Standard deviation
SRS: Surgical rating scale
TOF: Train-of-four

## References

[1] Buise M, Van Bommel J, Mehra M, Tilanus HW, Van Zundert A, Gommers D (2008). Pulmonary morbidity following esophagectomy is decreased after introduction of a multimodal anesthetic regimen. Acta Anaesthesiol Belg.

[2] Martini CH, Boon M, Bevers RF, Aarts LP, Dahan A (2014). Evaluation of surgical conditions during laparoscopic surgery in patients with moderate vs deep neuromuscular block. Br J Anaesth.

[3] Staehr-Rye AK, Rasmussen LS, Rosenberg J, Juul P, Gätke MR (2013). Optimized surgical space during low-pressure laparoscopy with deep neuromuscular blockade. Dan Med J.

[4] Williams MT, Rice I, Ewen SP, Elliot SM (2003). A comparison of the effect of two anaesthetic techniques on surgical conditions during gynaecological laparoscopy. Anaesthesia.

[5] Staehr-Rye AK, Rasmussen LS, Rosenberg J, Juul P, Lindekaer AL, Riber C (2014). Surgical space conditions during low-pressure laparoscopic cholecystectomy with deep versus moderate neuromuscular blockade: a randomized clinical study. Anesth Analg.

[6] García-Olmo DC, García-Rivas M, García-Olmo D (1998). Does neostigmine have a deleterious effect on the resistance of colonic anastomoses?. Eur J Anaesthesiol.

[7] Herz BL (1987). Colonic anastomotic disruption in myasthenia gravis. Report of two cases. Dis Colon Rectum.

[8] Bell CM, Lewis CB (1968). Effect of neostigmine on integrity of ileorectal anastomoses. Br Med J.

[9] Grosse-Sundrup M, Henneman JP, Sandberg WS, Bateman BT, Uribe JV, Nguyen NT (2012). Intermediate acting nondepolarizing neuromuscular blocking agents and risk of postoperative respiratory complications: prospective propensity score matched cohort study. BMJ.

[10] Kern SE, Johnson JO, Orr JA, Westenskow DR (1997). Clinical analysis of the flexor hallucis brevis as an alternative site for monitoring neuromuscular block from mivacurium. J Clin Anesth.

[11] Rex C, Wagner S, Spies C, Scholz J, Rietbergen H, Heeringa M (2009). Reversal of neuromuscular blockade by sugammadex after continuous infusion of rocuronium in patients randomized to sevoflurane or propofol maintenance anesthesia. Anesthesiology.

